# Over‐the‐counter bite splints: A randomized controlled trial of compliance and efficacy

**DOI:** 10.1002/cre2.315

**Published:** 2020-08-10

**Authors:** Geoffrey Gerstner, Wei Yao, Krishnapriya Siripurapu, Hadel Aljanabi, Ann Decker, David Ludkin, Rachel Sinacola, Katherine Frimenko, Kathryn Callaghan, Sean Penoyer, Claire Tewksbury

**Affiliations:** ^1^ Department of Biologic and Materials Sciences and Prosthodontics University of Michigan School of Dentistry Ann Arbor Michigan USA; ^2^ University of Michigan School of Dentistry Ann Arbor Michigan USA; ^3^ Department of Periodontics and Oral Medicine University of Michigan School of Dentistry Ann Arbor Michigan USA

**Keywords:** clinical efficacy, occlusal splints, sleep bruxism, treatment adherence

## Abstract

**Background:**

Occlusal splints are often used to curb the impacts of sleep bruxism (SB) on the dentition, and over‐the‐counter (OCT) options are becoming increasingly popular. OTC splints are usually fabricated at home by patients, but not routinely evaluated by dental professionals. It is unclear how OCT splints compare with more traditional splints that receive dental oversight.

**Objectives:**

The present randomized controlled study tested how an OTC splint compared with a gold standard bite splint in terms of patient compliance (primary outcome) and efficacy (secondary outcomes).

**Methods:**

Sixty‐seven subjects were randomly assigned to receive either the OTC (SOVA, N = 35) splint or the gold standard “Michigan” bite splint (MI, N = 32), with 61 completing the study (SOVA, N = 30; MI, N = 31). OTC‐splint subjects were required to fabricate their splints to clinically acceptable standards. Both groups wore the splints nightly for four months. Compliance was measured via daily diary. Efficacy outcomes evaluated stability, retention, periodontal health, night‐time rhythmic masticatory muscle activity (RMMA), and material wear.

**Results:**

OTC‐splint subjects had difficulty fabricating splints to clinically acceptable standards. The number of night‐time RMMA bursts was significantly greater for the OTC splint group. Compliance and all other efficacy measurements were not significantly different between‐groups.

**Conclusions:**

The results support the potential use of OTC splints for curbing the impacts of SB. However, the results strongly suggest that dentists should be actively engaged in overseeing patients' use of self‐fabricated appliances. This clinical trial is registered at ClinicalTrials.gov, Identifier number NCT02340663.

## 1 INTRODUCTION

Bite splints or occlusal appliances are often used to reduce tooth wear caused by sleep bruxism (SB), clenching and grinding (Cunha‐Cruz, Pashova, Packard, Zhou, & Hilton, 2010). They serve an important purpose, as tooth wear is a prevalent condition, which increases with age (Cunha‐Cruz et al., 2010). Terminating splint use can cause both temporomandibular disorder (TMD) and sleep bruxism (SB) symptom exacerbation (Rehm et al., 2012).

Three commonly recognized appliances exist (Maeda, Kumamoto, Yagi, & Ikebe, 2009): (a) an over‐the‐counter (OTC), non‐adjustable type, (b) an OTC, intra‐orally‐formed or “boil‐and‐bite” type, and (c) custom appliances. OTC devices are typically used without dental supervision, whereas the custom appliances involve dental intervention. Studies suggest that custom hard acrylic or boil‐and‐bite splints have nearly equal efficacy (Klasser, Greene, & Lavigne, 2010); however, evidence suggests that hard acrylic splints are superior to soft or repositioning appliances in managing TMD pain (Fricton et al., 2010).

Custom splints are usually expensive, and replacements are not typically covered by insurance. This is problematic, as splints can wear out with time (Korioth, Bohlig, & Anderson, 1998), despite long‐term splint use being necessary in bruxers (Rehm et al., 2012). OTC splints are becoming more popular, as they are inexpensive and convenient. However, few studies have evaluated OTC appliance efficacy and compliance. This is important, as it is unclear whether private practitioners evaluate OTC appliances routinely with their patients. This study's objective and purpose was to compare a specific custom appliance, the “Michigan bite splint,” to an OTC boil‐and‐bite appliance for compliance and measures of efficacy. By necessity, this was a short‐term trial; however, we attempted to evaluate many issues in that time interval. This is an FDA‐registered RCT, publically visible at: (https://clinicaltrials.gov/ct2/show/NCT02340663?cond=bruxism&draw=4&rank=38).

## 2 METHODS

### 2.1 Participants

**FIGURE 1:**
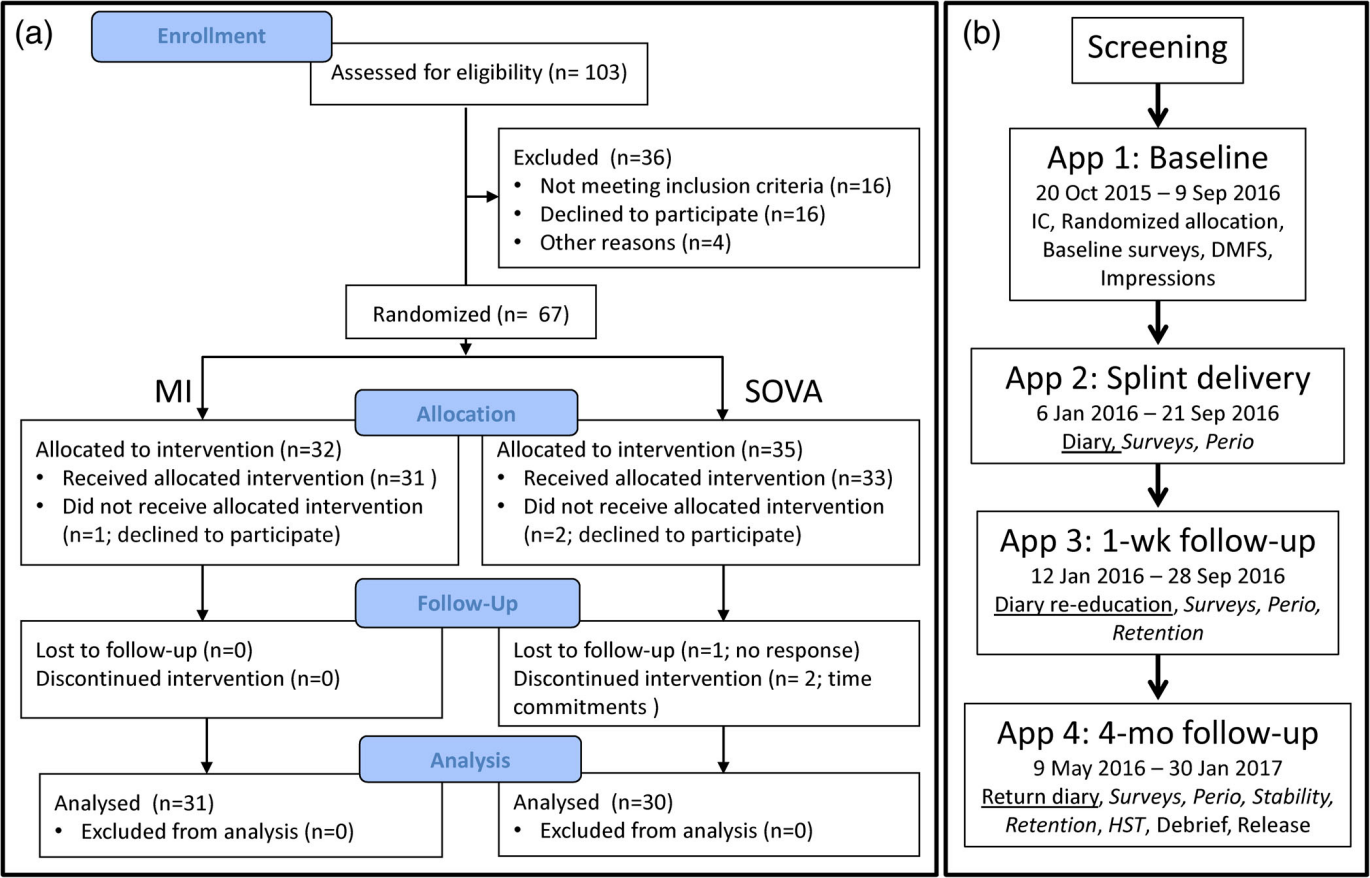
(a) Summary of participant numbers at selective time points during study. See Table 2 for demographic details. (b) Study sequence. Subjects meeting selection criteria were involved in four appointments. Time periods over which each appointment occurred are indicated. Primary outcome (Diary‐based compliance, underlined) was evaluated after app 4. Secondary outcomes (italicized) were evaluated during specific appointments, 2–4, as indicated. app, appointment; IC, informed consent; DMFS, decayed missing and filled surfaces; Perio, evaluation of gingiva and plaque indices; HST, home sleep testing (see text)

This randomized controlled study was approved by the University of Michigan Medical IRB (HUM00085489). Figure 1a shows recruitment and retention numbers. Subjects were recruited via announcements posted throughout the University of Michigan School of Dentistry. Screening occurred in the principal investigator's (PI, author GEG) laboratory between July 21, 2015 and September 2, 2016 and involved 103 candidates, 36 of whom did not meet inclusion–exclusion criteria, decided not to participate, or did not return calls. Subject losses occurred after randomized allocation (three subjects) or between Week 1 and the study's conclusion (three subjects). Thus, 30 SOVA and 31 MI subjects completed the study. This was considered sufficient, based upon a power analysis using SB data in Huynh, Manzini, Rompré, and Lavigne (2007). Using mean bruxing episodes per hour with an occlusal splint = 3.97 versus with a palatal splint = 4.45, a *SD* = 0.63, an *α* = .05 and a *β* = .8, we concluded a study would be sufficiently powered with 28 subjects group^−1^.

Inclusion criteria were: (a) ≥18 years old, (b) clinical signs of dental wear, (c) self‐report of nocturnal tooth grinding noises, (d) self‐report of a bruxing diagnosis by a family dentist, (e) absence of dental and medical conditions, including periodontal disease, cardiovascular disease, sleep apnea, movement, neurologic and sleep disorders, (f) full dentition sans third molars, (g) no active orthodontics nor removable prostheses, (h) no medications known to have movement disorder or sleep disturbance side effects, (i) ability to follow instructions, (j) no jaw function limitations, and (k) ability to report to the clinical laboratory at appointed times. Presence of TM joint noises or myalgia was permitted; however, joint arthritides were not.

**FIGURE 2:**
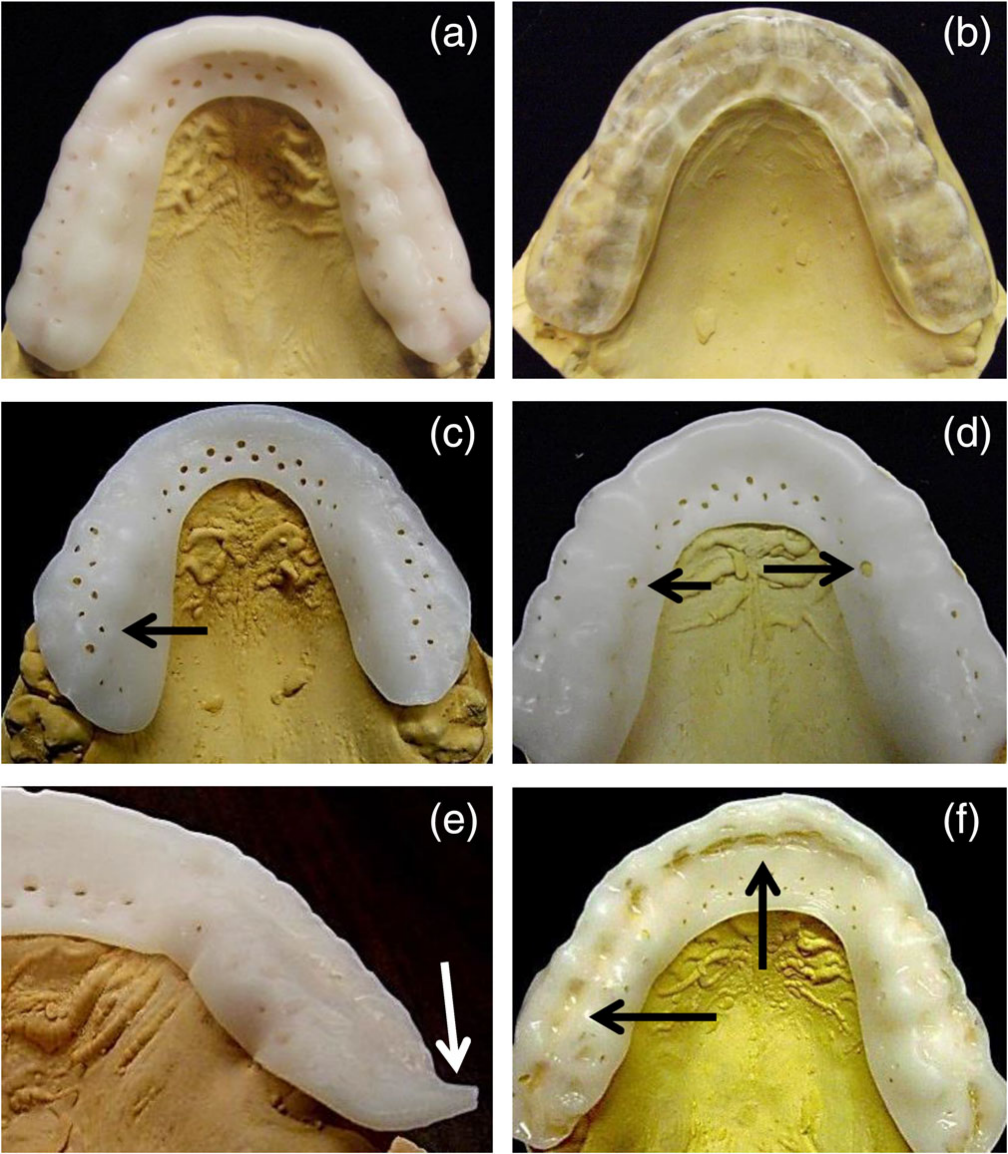
Occlusal views of the SOVA splint (a), and MI splint (b). Note the circular perforations in the SOVA splint. (c–f) Common SOVA splint fabrication errors (see text). (c) Rotation of splint. Perforations should be on buccal tooth surfaces, whereas non‐perforated portion of splint should be on occlusal surface. (d) Stretched material, evidenced by distorted perforation sizes (arrows). (e) Excessive material distal to second molar. (f) Excessive occlusal indentations from biting into splint during fabrication, despite instructions from manufacturer telling subjects not to bite into splint during fabrication

Consented subjects (App 1, Figure 1b) were randomly assigned to either an OTC “boil‐and‐bite” appliance group (SOVA, Akervall Technologies, Saline, MI; Figure 2a), or an acrylic occlusal appliance group, Michigan (MI) appliance (Figure 2b) using a stratified randomization procedure (Suresh, 2011), run in Excel (version 2010, Microsoft) with custom algorithms created by the PI. Groups were matched for gender and presence/absence of TMD signs/symptoms using the Diagnostic Criteria for TMD (TMD‐RDC; Dworkin & LeResche, 1992), with the TMD‐RDC performed by the PI or one other clinician, both of whom had extensive calibrated training in its use. Other instruments used included the Jaw Function Limitation Scale (JFLS; Ohrbach, Larsson, & List, 2008), TMD Pain Screener (Gonzalez et al., 2011), Measure of Symptoms Sleep Scale (MOS; Spritzer & Hays, n.d.), Perceived Stress Scale (PSS; Cohen, Kamarck, & Mermelstein, 1983), and Oral Behaviors Checklist (OBC; Markiewicz, Ohrbach, & McCall, 2006). All clinical components of the study were performed either in the Implant Clinic or the PI's clinical research laboratory, both in the University of Michigan's School of Dentistry.

Baseline intra‐ and extra‐oral exams performed by the PI evaluated dental and medical health. Alginate impressions were taken and poured in dental stone. The stone models were used to fabricate the MI appliance or to assess SOVA appliance fabrication (below).

Many of the methods described, below, appear in summary form in Table 1.

**TABLE 1:** Summary of methods

Assessment	Variable	How acquired	When	Metric
Bruxism grading	1. Bruxism frequency over 1‐month time period (nights/week) 2. Tooth wear	1. OBC, question 1 2. Intra‐oral exam and inspection of mandibular stl model	Screening	Per Reference (Lobbezoo et al., 2013), viz., 1. Ordinal Likert scale 2. Ordinal: 0 = no wear; 1 = wear into enamel; 2 = wear into dentine
Splint fabrication	Fabrication errors^a^	Examination of splint presented to PI as completed by subject	Appt 2	Categorical: Presence/Absence of an error; SOVA splints only
Compliance	1. Number of nights worn 2. % Total nights worn	Daily diary	Appt 4	1. Numeric 2. Percent
Ease of fabrication	Responses from SOVA subjects	Questionnaire	Appt 4	Ordinal Likert scale
User satisfaction	Responses from all subjects	Questionnaire	Appt 4	Ordinal Likert scale
OHIP, TSK, PS	Responses from all subjects	Questionnaires	Appts 1, 4	See References (Gonzalez, Schiffman, et al., 2011; Slade & Spencer, 1994; Visscher, Ohrbach, van Wijk, Wilkosz, & Naeije, 2010) Combinations of ordinal Likert scale and categorical data
Stability	Displacement of splint on dentition	2 × 5 Trials of each jaw‐movement task, EMG and jaw movement sensors	Appt 4	Millimeters
Retention	Self‐report of number of splint dislodgements	Five trials of eight orofacial movement tasks, self‐report	Appts 3, 4	Numeric, maximum of 40
Tissue health	1. Number of tooth surface areas with visible plaque 2. Severity of gingivitis	1. Intra‐oral exam with disclosing solution 2. Intra‐oral exam of marginal gingiva	Appts 2, 3, 4	Per References (Lobene, Weatherford, Ross, Lamm, & Menaker, 1986; Loe & Silness, 1963; Rustogi et al., 1992) 1. Numeric ratio 2. Mean ordinal grading
RMMA	1. Number of EMG bursts/hr 2. Number of EMG sequences/hr	Home sleep test	Appt 4	Quantitative ratios
Splint surface wear	Change in surface	Software algorithms applied to stl files	Appts 2, 4	Difference between stl models (appt 4 minus appt 2) in millimeters

Abbreviations: Appt, appointment; EMG, electromyography; OBC, Oral Behavior Checklist; OHIP, Oral Health Impact Profile; PI, principal investigator; PS, TMD Pain Screener; RMMA, rhythmic masticatory muscle activity; TSK, Tampa Scale for Kinesiophobia for TMD.

a Error categories included: (1) water bath temperature not correct, (2) splint not thoroughly warmed, (3) incisal bite not on anterior bar pad of splint blank, (4) lack of snugness against palate (>1 mm gap), (5) lack of snugness against facial/buccal tooth surfaces (<1 mm), (6) lack of sufficient material coverage on facials of anterior teeth, that is, flange is short of gum line, (7) material folded over on itself, (8) marks from lower dentition excessive, (9) material overstretched, that is, major axis > twice length of minor axis of perforations, (10) maximum intercuspation not even in clench, (11) splint falls off cast when inverted or shaken, (12) material orientation issues, viz., asymmetry/rotation, translated laterally or anteroposteriorly on occlusal surfaces, (13) distal of posterior‐most tooth not adequately covered, (14) posterior flange of splint extends onto soft tissues. Note: Because error categories were determined a priori, not all error categories were actually observed in the sample. Moreover, no additional error categories were observed or added post hoc.

### 2.2 Bruxism grading

**FIGURE 3:**
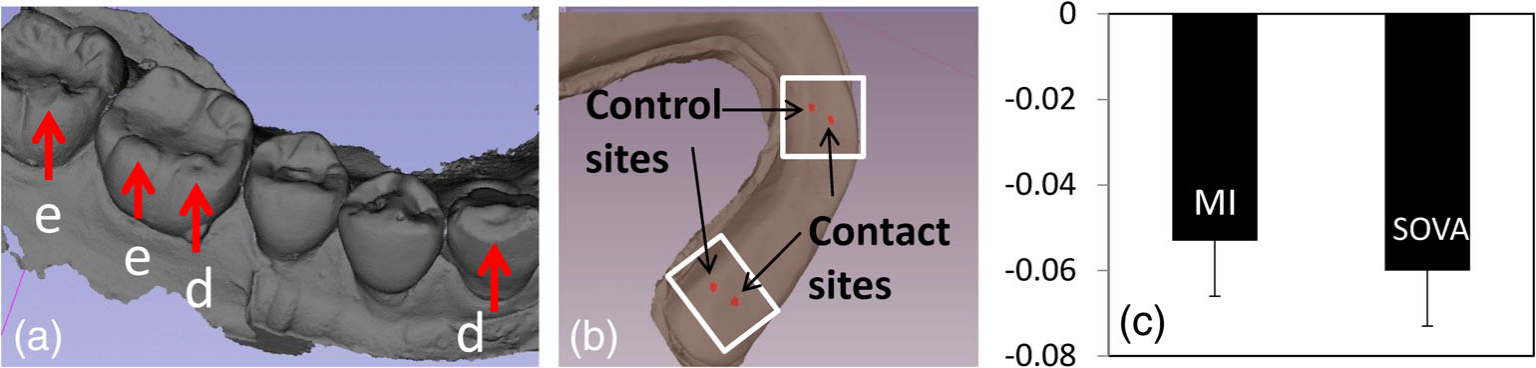
(a) Mandibular stl models were used for tooth wear grading, here showing examples of wear into enamel (e), which received scores of 1, and wear into dentin (d), which received scores of 2. (b) Splint stl model used for studies of material wear over the 4‐month time period. Rectangles show regions of interest used in assessments (see text). (c) Mean and 1 *SD* (error bars) of splint material wear for the MI versus SOVA group. Ordinate is distance (mm) reported as negative values to represent material loss

We used the international consensus criteria for grading bruxism in subjects (Lobbezoo et al., 2013). Question 1 of the OBC was used to define self‐reported bruxism. Bruxing signs were clinically evaluated, and confirmed on mandibular stereolithography (stl) models (Figure 3a; True Definition Scanner, 3M, St. Paul, MN). Scores of no wear (0), wear into enamel (1), and wear into dentin (2) were assigned to each tooth, based upon severest detected wear on each tooth, and subjects were scored using the median value for the mandible. Scoring and grading were done by an investigator blinded to group assignments.

### 2.3 Splint delivery

SOVA subjects fabricated SOVA devices in the clinic during Appointment 2 (Figure 1b) using manufacturer instructions. If they felt compelled to call the company's help line, they instead asked the PI, who was versed in the company's help line scripts. Otherwise, subjects fabricated splints without oversight. Once fabricated, splints were assessed for critical errors (see footnote, Table 1, for description of error categories). Critical errors required subjects to refabricate splints to acceptable standards. Total refabrications, total time spent fabricating, and types of critical errors were scored.

The MI appliance was fabricated in a professional dental laboratory routinely used by the UM School of Dentistry for fabrication of bite splints. The MI appliance was fabricated by the lab between Appointments 1 and 2 (Figure 1b). At delivery, MI splints were adjusted for fit and to establish bilateral occlusal contacts.

### 2.4 Compliance (primary outcome)

Daily diaries were filled out at home over the 4‐month study period. Instructions stressed that the diaries be filled out daily. Subjects were instructed to account accurately for nights the splints were not worn. Compliance was defined by the total number of nights of splint wear and the percent of total nights worn (Table 1). Diaries were assessed at Week 1 to determine whether subjects were correctly reporting compliance and to re‐educate as necessary. The null hypothesis was that no significant differences in number of nights of splint wear nor in percent of nights worn would exist between groups.

### 2.5 Surveys

SOVA subjects completed a survey covering ease of fabrication. At study's end, all subjects completed a survey on user satisfaction. Standard surveys used at Appointments 1 and 4 to assess changes over time included the Oral Health Impact Profile (OHIP; Slade & Spencer, 1994), Tampa Scale for Kinesiophobia for TMD (TSK; Visscher et al., 2010), and TMD Pain Screener (PS; Gonzalez, Schiffman, et al., 2011). No hypothesis was formalized for ease of fabrication; however, we anticipated that SOVA subjects would report that splints were easy to fabricate. Null hypotheses for other surveys were: (a) User satisfaction with splints will not be significantly different between subject groups. (b) There will be no significant between‐group differences in responses on the three standard surveys at baseline nor at study's end, nor will there be differences in the survey responses through time.

### 2.6 Efficacy (secondary outcomes)

**FIGURE 4:**
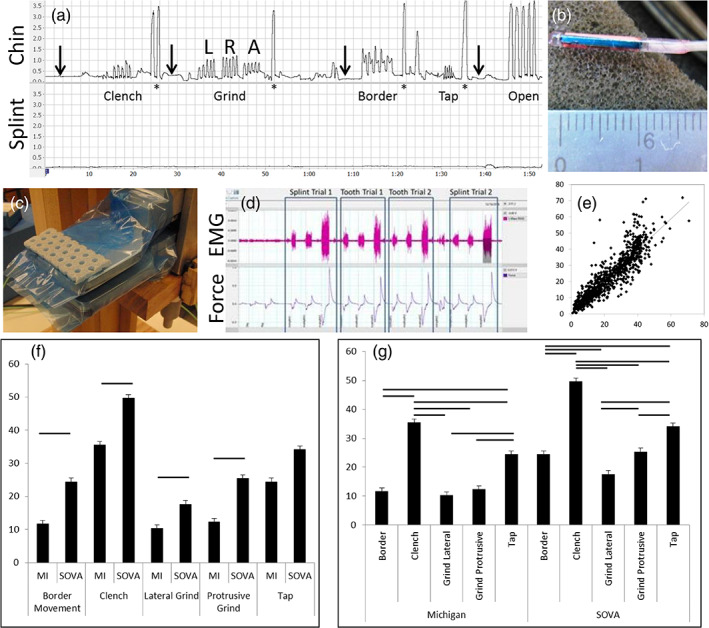
Stability testing methods. (a) Example of a trial showing chin (upper trace) and splint (lower trace) movements. Abscissa is time (min:s); ordinate is distance (shown in cm). Splint data were sampled during the time windows corresponding to each task identified by chin movements. Arrows identify time periods used to construct data for the “Rest” category (see text and Table 4). Letters L, R, A over the Grind trial indicate left, right, and anterior grinding, respectively. Border refers to a task where subjects' swept the jaw out to the facial border of the splint by moving the jaw first left laterally, then anteriorly, then right laterally and back to a rest position. (b) Close‐up of a microsensor used for tracking chin and splint movements; outputs from two such sensors resulted in the time series shown in (a). (c) Picture of the bite plate used to sample bite force data. The shown perforated thermoplastic was used to provide contact with first molar regions bilaterally, with and without bite splints in place. With thermoplastic in place, vertical dimension was 20 mm and was not adjusted for trials with and without the splints in place. (d) Example of a bite force trial. Upper trace is right masseter EMG; lower trace is output from the force transducer. Rectangles partition trials into four replicates, each of which involved mild, moderate and high bite forces in sequence. The four replicates were: splint in, splint out (tooth trial), splint out, splint in, in that order for all subjects. (e) Scatter plot of actual bite force (abscissa) against EMG‐based estimate of bite force (ordinate). Both axes are in kg. Plotted data are from the final three trials, one with splints in place and two trials with splints removed, with each trial including mild, moderate and high bite forces as shown in (d). (f and g) Mean (*SD*) bite force estimates by group and task. Ordinate in both plots is estimated bite force (kg). Horizontal bars indicate pairwise comparisons that were statistically significant, Bonferonni‐corrected at the *p* < .05 level. Note that left and right lateral components of the Grind task were pooled to create a Lateral Grind category whereas the anterior component of Grind is Protrusive Grind (see also Table 4, which reports splint displacements in mm during the tasks). The lateral and protrusive components are separated in order to report results for “roll” and “pitch” degree‐of‐freedom dislodgements independently

Efficacy was defined by stability, retention, periodontal health, and estimated RMMA (Table 1). Stability was tested at Appointment 4 (Figure 1b) to assess splint dislodgement on the maxilla. Five tasks were performed in the following order (Figure 4a): clench; grind to the left, right and anteriorly; move the jaw to the left, forward, around to the right, and back to a biting position (Border); tap; open wide. Each task was performed five times in a row (trial, Figure 4a), and two such trials were performed, resulting in ten observations per task. A >10‐s rest period occurred between tasks. The null hypothesis was that there would be no significant between‐group differences in splint displacements (measured in mm in three‐dimensional space) caused by the tasks.

*Stability* was monitored using a magnetometer motion analysis system and 1.8 micro sensors attached to the chin and splint (Figure 4b, Liberty, Polhemus, Colchester, VT). If the splint was dislodged during tasks, the splint sensor recorded this in three dimensions. The maximum change in this distance per task was used to assess stability.

Subjects were asked to bite hard during tasks, and bite force estimates were made to confirm this. Prior to performing tasks, subjects bit on a custom bite plate (Figure 4c) connected to a force sensor (Kistler 9203 force sensor ±500 N, Kistler 5010 Dual Mode Amplifier); the design was based on Herrel, Spithoven, van Damme, and De Vree (1999), with modifications to standardize the bite position to the first molar region. While subjects bit on the transducer, surface electromyography (EMG) was sampled from the masseter muscles bilaterally at 10 KHz and filtered with a 20–500 Hz bandpass filter (Ag/AgCl electrodes, MVAP‐II, MVAP Medical Supplies, Newbury Park, CA; Powerlab 8/35, Octal Bio Amp, and LabChart 8, AD Instruments, Colorado Springs, CO).

Subjects bit with mild, medium, and maximum forces, twice with the splint in and twice with the splint out. The bite plate thickness was standardized to 20 mm. A 10‐s rest period occurred between bites. An example of one such trial is shown in Figure 4d.

The filtered right masseter EMG bursts were used to extract root mean square (RMS), the mean and median power frequencies, and peak amplitude, expressed as a percent of the maximum peak amplitude (LabChart 8, AD Instruments, Colorado Springs, CO). A step‐wise linear regression, with a procedure to eliminate variables expressing high collinearity, was used to create an equation to estimate bite forces. The first “splint‐in” bite force trial was used as the training set; the remaining three trials served as test sets.

Test‐set results (Figure 4e) were evaluated for precision and accuracy with Lin's concordance coefficient, ρ_c_ (Lin, 1989) and the bias correction factor, C_B_ (McBride, 2005), respectively. Results indicated moderately strong precision (ρ_c_ = 0.9013, 95% CI = 0.89–0.91) and high accuracy C_B_ = 0.9979), suggesting that averaging bite force estimates would improve precision; hence, mean estimates were used in analyses.

*Retention* was assessed during Appointments 3 and 4 (Figure 1b, Table 1) via self‐report while subjects performed eight tasks: forced blowing; lip and cheek shearing movements to attempt to unseat the splint; coughing; yawning; smiling; swallowing; sucking and puckering; maximum openings. Each task was repeated five times. Between tasks, subjects bit gently to determine whether splint reseating occurred, in which cases subjects reported a positive score. Total positive observations were tallied (maximum score = 40, 8 tasks × 5 replicates per task). The null hypothesis was that no significant between‐group differences would exist in number of reported splint reseatings following tasks at either Appointment 3 or 4.

**FIGURE 5:**
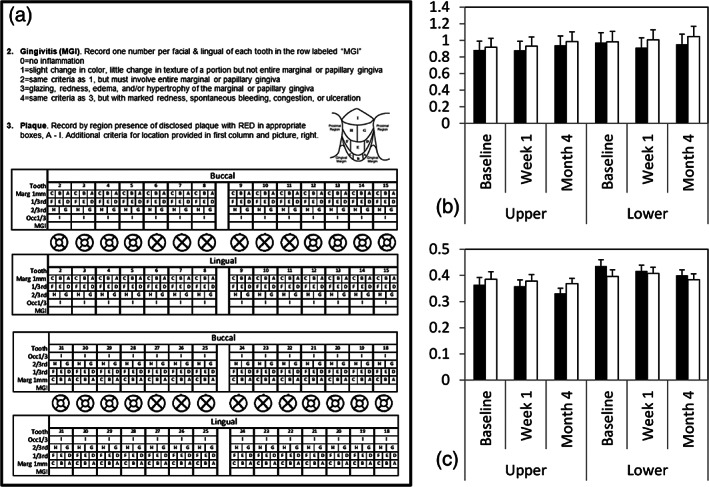
(a) Page from laboratory manual used by periodontal investigators to measure gingivitis (MGI) and plaque index (PI). Page included instructions and scoring charts as shown. (b) Mean (1SD) marginal gingiva indices (MGI) for the Michigan group (filled) and SOVA group (unfilled) at baseline, after 1 week and 4 months with splints. Upper = upper arch; lower = lower arch. (c) Mean (1SD) plaque indices (PI); format similar to MGI plot

*Tissue health* was assessed using the Rustogi modification of the Navy plaque index (PI; Rustogi et al., 1992) and the modified gingival index (MGI; Lobene et al., 1986; Loe & Silness, 1963; Figure 5a, Table 1). Assessments were done by calibrated periodontal residents in the UM School of Dentistry, who were blinded to group assignments. Each resident assessed equal numbers of subjects from each group to minimize scoring biases. Periodontal data were taken at Appointments 2, 3 and 4 (Figure 1b). PI and MGI scores were calculated separately for upper and lower arches and also for the buccal and lingual of the upper and lower arches. The null hypothesis was that there would be no significant between‐group differences in PI or MGI, nor would there be time‐dependent effects.

**FIGURE 6:**
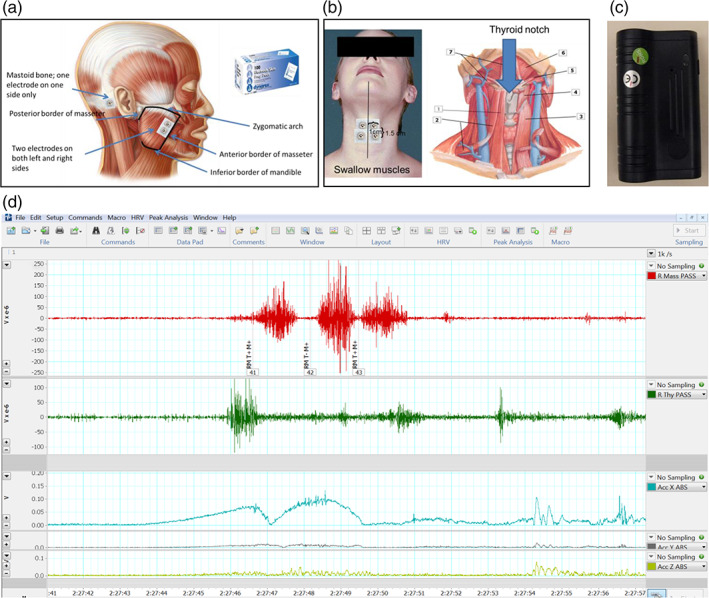
Top row shows instructions from the laboratory manual used for calibrated placement of masseter (a) and thyroideus (b) EMG electrodes. Also shown is the audio monitor (c) used to monitor room and subject sounds, see text. Bottom row (d) shows an example of ~15 s from a subject's home sleep study. Top trace is right masseter, middle trace is right thyroideus and bottom three traces are body movements. Note the sequence of three masseter bursts, representing an RMMA sequence. Note that this sequence was associated with thyroideus activity and body movements

Finally, RMMA was estimated (BioRadio Recording Unit, Great Lakes NeuroTechnologies, Valley View, OH) in a home sleep study performed at Appointment 4 (Figure 1b and Table 1). Bipolar EMG electrodes (Ag/AgCl, MVAP‐II, MVAP Medical Supplies, Newbury Park, CA) were placed bilaterally on the masseter and thyroideus muscles (Figure 6a,b). A ground electrode was placed on the mastoid process opposite the subject's sleeping side preference (Figure 6a). Body movements and positions were also sampled. Audio was used to assist with identifying bruxing events and to identify other nocturnal noises, for example, coughing, talking, and so on (Figure 6c). The monitor was worn on an upper arm of the subject's choosing.

Data were recorded at 1 kHz, filtered (EMG, 20–500 Hz bandpass; body movements, 20 Hz low pass), and evaluated in 10‐s epochs by investigators trained and calibrated to analyze recordings using published criteria (Carra, Huynh, & Lavigne, 2012). Scorers were blinded to subject group assignment, and they received equal numbers of subjects from each group to reduce rater bias.

Gold standard evaluation of SB uses polysomnography (PSG) and audio–video capture (Lobbezoo et al., 2013); however, home monitoring is an acceptable alternative (Ahlberg, Savolainen, Paju, et al., 2008), despite moderate false positive rates (Carra, Huynh, & Lavigne, 2015). Also, RMMA is not necessarily SB, and without EEG and EOG leads, recording time is not differentiable from sleep time. Thus, this study, *sensu stricto*, reports RMMA during total recording time; however, to reduce the false positive rate, three trained investigators used the monitor at home and performed the following tasks while in bed.

*Oral behavior artifacts*: swallow; lick lips; speak; moan; snore; cough; yawn; open wide; laugh; RMMA without tooth contact; rapid light tooth tapping. Each task was repeated five times.

*Body movement artifacts*: rise on elbows; scratch arms; reposition legs; move legs rhythmically; move from prone to left and right sides, and from supine to left and right sides; move head from left to right cheek and reverse while prone. Each task was repeated five times alone, then in conjunction with bruxing‐like RMMA, below.

*Bruxing‐like RMMA*: clench for 3 s (tonic clench); clench rhythmically five times at 1 Hz (phasic clench); grind rhythmically five times at 1 Hz to the right and to the left (phasic grind). Each RMMA was repeated five times without and then with the body movement artifacts. Investigators were trained to avoid movements other than those called for by each task.

Recorded data were processed and scored similar to data obtained from subjects. All scored observations were matched to their true task categories for cross‐validation.

Scored EMG bursts from each investigators' data set were subjected to a step‐wise discriminant analysis. Each investigator's resulting functions were cross‐validated on the data from the other two investigators. We determined that true positive and negative rates could be reduced if we required agreement from two raters' equations; this generated a true positive rate of 88% and true negative rate of 91%. Thus, positive categorization by at least two raters' equations was used to define estimated RMMA in subject data. The null hypothesis was that no between‐group differences would exist in RMMA, tested as both number of EMG bursts and as number of EMG episodes.

To assess *splint surface wear*, polyvinyl‐siloxane (PVS) impressions of the splint were obtained during Appointments 2 and 4. Impressions were poured in stone (Diamond Die Scan Stone, Hi‐Tech Dental Products, Greenback, TN) and digitized (3M True Definition Scanner). Mesh files (Figure 3b) were uploaded into 3D Slicer (Version 4.8.1 www.slicer.org (Fedorov et al., 2012)) and registered (CMFreq extension of 3D Slicer). Two regions of interest (ROI) were registered, one around first molar‐splint contacts, and one around canine‐splint contacts (Figure 3b, rectangles). The left side was analyzed unless clearer signs of wear occurred on the right side.

The Model‐to‐Model Distance extension in 3D Slicer was used to calculate the signed closest point distances between the aligned surfaces of the baseline and 4‐month‐old stl models. The average distance (in mm) was calculated at two specific sites within each ROI, one site where tooth‐splint contact occurred (contact sites) and an adjacent site where no tooth‐splint contact could occur (control sites; Figure 3b). Means for control and test sites were obtained using areas with five‐voxel radii (Pick 'n Paint extension in 3D Slicer). Mean distances between splint surfaces were calculated (Mesh Statistics extension in 3D Slicer). Estimated wear, w, was calculated as w = d_control_ − d_test_, where d_test_ = mean between‐model distance at the tooth contact site, and d_control_ = mean between‐model distance at the control site (Table 1). The null hypothesis was that no differences in splint material wear will exist between subject groups.

### 2.7 Statistical tests

Continuous data were evaluated for normality using Q–Q plots, and tests for skewness and kurtosis. If normality assumptions were violated, data were transformed. If normality was not achieved through transformation, we used non‐parametric tests. General linear models (GLM) were used for tests of within‐ and between‐group differences. A repeated measures design was used for data collected multiple times during the study. Pearson's product moment was used on normally distributed data.

For non‐normally distributed data and non‐continuous data, the Mann–Whitney *U* test was used for comparisons between groups. For tests involving more than two categories, Kruskall–Wallis or Friedman tests were used. The Wilcoxon or Friedman test was used for paired tests. For correlation analyses of non‐continuous data, Spearman's rank correlation test was used.

Because the study's aim was to determine whether the SOVA splint was similar to the MI splint, we employed a liberal, non‐corrected *p* < .05, except where GLM post‐hoc tests were performed, in which case, a Bonferroni correction was used. Effect sizes and 95% CI were calculated for primary and secondary outcomes (www.psychmetrica.de/effect_size.html, www.effect‐size‐calculator.herokuapp.com/#form4).

## 3 RESULTS

Table 2 shows initial and final enrollment demographics. There were no significant differences between the two groups. No between‐group differences existed in self‐report of SB (MWU = 417, *p* = .462). All subjects reported current SB noises and showed clinical signs of wear (5 had scores of 1; 5 had scores of 1.5; 51 had scores of 2). Twenty‐five subjects (11 SOVA, 14 MI) reported bruxing 4–7 nights/week, 26 subjects (12 SOVA, 14 MI) reported bruxing 1–3 nights/week, and nine subjects (6 SOVA, 3 MI) reported 1–3 nights per month. (Data for one remaining SOVA subject spanned multiple categories).

**TABLE 2:** Demographics

	SOVA	MI	Total
*Initial enrollment*			
N	35	32	67
Age	25.5 (3.30)	25.5 (3.82)	25.5 (3.53)
Gender (F:M)	19:16	15:17	34:33
Ethnicity			
Amerind	0	1	1
Asian	7	5	12
Native Hawaiian; Pacific Islander	0	0	0
Black/African American	2	2	4
White/Caucasian	26	23	49
>1 race	0	1	1
Unknown/unreported	0	0	0
Hispanic/Latin	3	1	4
Not Hispanic/Latin	0	0	0
Unknown/not reported	0	0	0
*Final enrollment*			
N	30	31	61
Age	25.0 (2.91)	25.1 (3.25)	25.0 (3.06)
Gender (F:M)	16:14	15:16	31:30
Ethnicity			
Amerind	0	1	1
Asian	6	4	10
Native Hawaiian; Pacific Islander	0	0	0
Black/African American	2	2	4
White/Caucasian	22	23	45
>1 race	0	1	1
Unknown/unreported	0	0	0
Hispanic/Latin	2	1	3
Not Hispanic/Latin	0	0	0
Unknown/not reported	0	0	0

*Note*: Age: Initial enrollment, *p* = .932; final enrollment, *p* = .903. Gender: Initial enrollment, *p* = .547; final enrollment *p* = .702. Ethnicity: Initial enrollment, *p* = .545; final enrollment, *p* = .302.

No significant differences in tooth wear existed between groups (MWU = 415, *p* = .537). Sixty subjects had wear facets on all posterior teeth; one remaining case (SOVA) had wear facets into enamel on all molars. A trend existed between clinical wear assessment and bruxing habit self‐report scores (Spearman's ρ = 0.248, *p* = .059, df = 59).

No significant between‐group differences existed for TMD by category (Table 3). Also, there were no significant between‐group differences in overbite, overjet, maximum pain‐free opening, maximum voluntary opening, maximum opening with passive stretch, maximum protrusion or maximum left or right laterotrusions (Table 3).

**TABLE 3:** TMD, periodontal, and survey results

TMD	SOVA	MI	Statistical test
	*N*	*N*	MWU, *p*
No symptoms	16	12	397, .26
Myalgia	10	5	416, .32
Headache due to myalgia	9	8	453, .82
Arthralgia	0	1	465, 1.0
DD with reduction	11	15	364, .083
DD with reduction, intermittent locking	0	2	435, .16
DD No reduction, limited opening	0	0	–
DD No reduction, no limited opening	0	0	–

	Mean (*SD*)	Mean (*SD*)	F(1,59), *p*
Pain‐free opening	50.2 (6.4)	51.7 (8.8)	0.6, .4
Voluntary opening	55.1 (5.6)	57.2 (7.7)	1.5,.2
Open with passive stretch	57.2 (5.1)	58.8 (7.2)	1.0, .3
Protrusion	8.4 2.3	8.8 2.4	0.6, .4
Left laterotrusion	9.7 2.2	10.1 2.2	0.6, .4
Right laterotrusion	9.2 1.8	9.6 2.0	0.7, .4

Survey results	Mean (*SD*)	Mean (*SD*)	MWU, *p*
JFLS			
Masticatory function	0.61 (0.95)	0.59 (0.78)	457.5, .91
Mobility	0.41 (0.93)	0.42 (0.81)	427.5, .69
Verbal and communication	0.05 (0.19)	0.03 (0.11)	462.5, .95
Total	0.35 (0.51)	0.34 (0.45)	438.5, .86
MOS			
Sleep disturbance	27.4 (21.21)	24.7 (20.43)	426, .57
Snoring	19.3 (20.67)	18.7 (27.29)	423.5, .52
Sleep short of breath or headache	5.3 (10.42)	9.7 (18.53)	420.5, .41
Sleep adequacy	48.3 (23.21)	46.1 (19.44)	440, .72
Sleep somnolence	36.7 (16.68)	40.0 (21.84)	436, .67
Sleep Problems Index 1	30.4 (15.38)	32.3 (12.86)	414.5, .47
Sleep Problems Index 2	32.4 (15.52)	34.0 (13.19)	420.5, .52
Optimum sleep	0.6 (0.50)	0.6 (0.51)	441, .69
PSS	13.37 (6.89)	12.23 (6.38)	491, .51
OBC			
Raw sum	26.7 (7.01)	30.8 (8.44)	328.5, .05
Sum of positive scores	13.37 (2.85)	14.23 (2.65)	381, .22

OHIP			F(1,59), *p* ^a^
Initial functional limitation scale	5.4 (1.1)	5.5 (0.5)	Tx: 0.16, .69
End functional limitation scale	5.6 (0.4)	5.3 (1.1)	Time: 0.006, .96
			Tx*Time: 2.0, .16
Initial physical pain scale	4.7 (1.0)	4.9 (0.6)	Tx: 0.47, .49
End physical pain scale	4.9 (0.6)	4.9 (1.0)	Time: 0.18, .68
			Tx*Time: 0.84, .36
Initial psychological discomfort scale	5.3 (1.1)	5.5 (0.7)	Tx: 0.10, .76
End psychological discomfort scale	5.5 (0.5)	5.4 (1.1)	Time: 0.07, .79
			Tx*Time: 0.99, .33
Initial physical disability scale	5.5 (1.1)	5.8 (0.3)	Tx: 0.01, .93
End physical disability scale	5.9 (0.3)	5.6 (1.1)	Time: 0.37, .54
			Tx*Time: 2.3, .14
Initial psychological disability scale	5.3 (1.1)	5.7 (0.4)	Tx: 1.0, .31
End psychological disability scale	5.5 (0.6)	5.5 (1.0)	Time: 0.08, .78
			Tx*Time: 2.8, .10
Initial social disability scale	5.6 (1.1)	5.9 (0.3)	Tx: 0.17, .68
End social disability scale	5.9 (0.3)	5.7 (0.9)	Time: 0.01, .92
			Tx*Time: 3.1, .08
Initial handicap scale	5.7 (1.1)	5.9 (0.2)	Tx: 0.03, .85
End handicap scale	5.9 (0.2)	5.7 (1.1)	Time: 0.01, .93
			Tx*Time: 2.9, .10
Initial total scale	5.3 (1.0)	5.6 (0.3)	Tx: 0.09, .76
End total scale	5.6 (0.3)	5.4 (1.0)	Time: 0.07, .79
			Tx*Time: 2.3, .14
Initial TSK	34.2 (6.2)	33.5 (6.3)	Tx: 0.30, .58
End TSK	33.4 (4.7)	32.8 (5.3)	Time: 0.90, .35
			Tx*Time: 0.001, .98

			*K‐W*, *p*
Initial TMDPS	2.0 (2.21)	2.4 (2.00)	Initial: 0.764, .39
End TMDPS	1.8 (1.87)	2.1 (1.88)	End: 0.49, .49

			Friedman, *p*
			Time: 1.95, .39

Abbreviations: K‐W, Kruskall–Wallis; MWU, Mann–Whitney U; p, *p* values; SD, standard deviation; Tx, treatment.

^a^ Results are effects due to treatment (tx), time, and interactions (treatment*time).

Baseline results for JFLS (Ohrbach et al., 2008), PSS (Cohen et al., 1983), OBC (Markiewicz et al., 2006), and MOS (Spritzer & Hays, n.d.) revealed no significant between‐group differences (Table 3). Three surveys, the OHIP (broken into subcategorized as per (Slade & Spencer, 1994)), TSK (Visscher et al., 2010), and TMDPS (Gonzalez, Schiffman, et al., 2011) were filled out at Appointments 1 and 4 (Initial, End, respectively, in Table 3). There were no differences between groups, nor through time, nor were there interactive effects in survey results.

Twenty SOVA subjects (66.7%) fabricated splints without asking for help, eight (26.7%) asked for help once, and two (6.7%) asked twice. Twenty‐eight (93.3%) subjects remolded splints prior to clinical inspection. Mean (SD) fabrication time was 14.0 (11.6) minutes.

Twenty‐seven (90%) splints had critical errors. Poor splint alignment (Figure 2c) on the occlusal table occurred in 26 (86.7%) splints. Other errors included over‐stretched material (N = 8, 26.7%, Figure 2d), inadequate or excessive posterior coverage on the posterior‐most molars (N = 19, 63.3%, Figure 2e), or excessive mandibular occlusal indentations (N = 6, 20%, Figure 2f).

Table 4 shows results for user satisfaction, composed of four items based upon a five‐point Likert scale; 1 = Strongly Disagree and 5 = Strongly Agree. No significant differences existed between groups.

**TABLE 4:** Subject satisfaction, stability, retention and estimated RMMA activity results

Independent variable	MI	SOVA	Test	Effect size	95% CI
Satisfaction	Mean (*SD*)	Mean (*SD*)	MWU		
The splint fits well	4.3 (0.8)	4.1 (1.2)	444.5	0.20^b^	−0.31–0.70
I do not use the splint nightly	1.8 (1.2)	2.2 (1.4)	389	0.31^b^	−0.20–0.81
I use an additional splint	1.5 (1.1)	1.6 (1.3)	442.5	0.08^b^	−0.42–0.59
The splint helps my bruxism	4.0 (0.9)	3.7 (1.0)	377.5	0.32^b^	−0.19–0.82

Stability^a^	Mean (*SD*)	Mean (*SD*)			
Clench	0.71 (1.58)	0.72 (1.94)			
Grind	0.78 (0.70)	0.83 (1.66)			
Border	1.12 (1.33)	0.63 (1.24)			
Tap	0.30 (0.36)	0.33 (0.41)			
Open	1.60 (1.30)	1.16 (0.94)			
Rest	0.16 (0.18)	0.22 (0.23)			

			F(1,58)		
Total	0.78 (1.14)	0.65 (1.16)	1.13	0.03^c^	0–0.13

*Retention*	*N*	*N*	*MWU*		
Wk 1					
0	23	21	391	0.28^c^	−0.22–0.78
1	7	7			
2	1	2			
3	0	0			
4	0	0			
>4	0	0			
Mo 4					
0	25	21	386	0.30^c^	−0.21–0.80
1	1	2			
2	3	0			
3	1	0			
4	0	1			
>4	1	6			

			*z*		
Wk 1 vs. Mo 4	31	30	−0.629	0.16^c^	−0.34–0.66

Estimated RMMA	Mean (*SD*)	Mean (*SD*)	F(1,51)		
N_B_	120.1 (124.0)	187.3 (147.2)	2.67	0.05^c^	0–0.20
N_E_	58.9 (47.0)	82.3 (51.3)	1.89	0.04^c^	0–0.17
Bh^−1^	18.8 (17.3)	31.2 (21.9)	4.24*	0.08^c^	0–0.24
Eh^−1^	9.4 (6.2)	13.7 (7.8)	3.32	0.06^c^	0–0.21
BE^−1^	1.7 (0.58)	2.2 (0.89)	4.90*	0.09^c^	0–0.25
T_B_	1.1 (0.52)	1.0 (0.33)	0.33	0.01^c^	–^d^
T_E_	2.3 (1.45)	2.9 (1.43)	1.47	0.03^c^	0–0.16
N_PE_	6.5 (9.1)	12.2 (12.0)	2.81	0.05^c^	0–0.20
N_TE_	9.5 (9.0)	10.1 (9.4)	0.01	0.00^c^	–^d^
N_ME_	8.3 (10.4)	11.9 (10.8)	0.99	0.99^c^	–^d^

Abbreviations: CI, confidence interval; SD, standard deviation; MWU, Mann–Whitney U; RMMA, rhythmic masticatory muscle activity; N_B_, number of EMG bursts; N_E_, Number of episodes; Bh^−1^, Bursts per hour; Eh^−1^, Episodes per hour; BE^−1^, Bursts per episode; T_B_, Burst duration; T_E_, Episode duration; N_PE_, Number of phasic episodes; N_TE_, Number of tonic episodes; N_ME_, Number of mixed episodes; z, Wilcoxon z‐score.

a Differences, in mm, across tasks were significant, F(5,290) = 52.389, *p* < .001, partial η^2^ = 0.475 (95%CI: 0.39–0.53); no task*group interactions F(5,290) = 1.486, *p* = .194, partial η^2^ = 0.025 (95%CI: 0–0.054).

b d_Cohan_ effect size.

c Partial η^2^ effect size.

d CI undefined (not calculable).

* *p* < .05.

Table 4 also shows stability test results. An example of a stability test is shown in Figure 4a. The table reports maximum dislodgements in mm by task, expressed as group means and 1 *SD*. Movement data for one SOVA subject was corrupted and not included. No significant between‐group differences existed. Opening wide produced the greatest dislodgement for both groups. Rest resulted in minimal dislodgement. No significant between‐group differences occurred (see also Table 4 footnotes for further results).

Bite force estimates during stability tasks are shown in Figure 4f,g. Horizontal error bars show significance at the *p* < .05 level. Estimated bite forces were higher among SOVA than MI subjects (F[1,118.6] = 12.604, *p* = .001, partial η^2^ = 0.096, 95% CI = 0.019–0.20; Figure 4f). Clenching produced the highest forces, whereas border and grinding produced lower forces for both groups (Figure 4g). No interactive effects were found.

Retention results, Table 4, are the number of dislodgements (Column 1) reported by number of subjects, N, (Columns 2 and 3). The maximum number of dislodgements that could have occurred = 40, but no splint had >6 dislodgements. No significant between‐group differences existed at Week 1 or Month 4, and results did not change significantly over the 4‐month study (Week 1 vs. Month 4 in table).

Finally, Table 4 shows estimated RMMA by group. Variables are defined using nomenclature from Carra et al. (2012). Three MI and five SOVA subjects' data were not analyzed due to recording errors. Total recording time (mean, *SD*) for both groups was 6.3 (1.6) hr for MI, 6.5 (1.4) hr for SOVA, t = −0.4, *p* = .70. Blinded raters participated in data analysis; rater effects were not significant (Wilk's lambda = 0.582, F[15,33] = 1.583, *p* = .113, partial η^2^ = 0.418, 95% CI = 0–0.414). A MANOVA indicated no significant effects due to splint group (Wilk's lambda = 0.688, F[15,33] = 0.997, *p* = .481, partial η^2^ = 0.312, 95% CI not calculable). However, note that SOVA had significantly more Bh^−1^ and more BE^−1^ than MI. There were no significant differences for other SB variables between groups.

**FIGURE 7:**
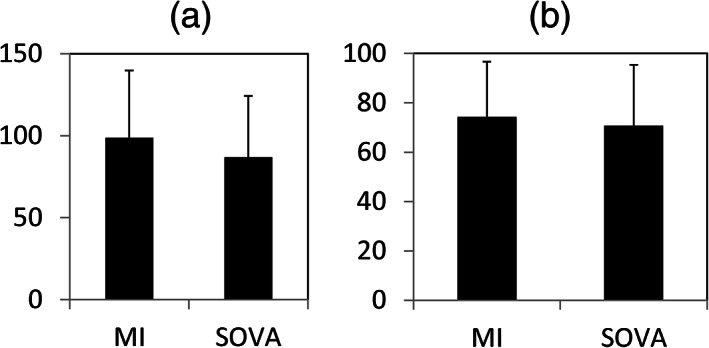
Compliance results plotted as total nights of splint wear (a), and as percent total days splint was in possession of subject (b). Histograms are means with 1 *SD* error bars for the MI versus SOVA groups

Figure 7 shows compliance results, both as the number of nights worn (a) and as percent of total days the splints were possessed (b). There were no significant differences in compliance (for number of nights worn, t = 1.201, df = 62, *p* = .233, d_cochran_ = 0.308, 95% CI = −0.20–0.81; for percent nights worn, t = 0.602, df = 62, *p* = .549, d_cochran_ = 0.154, 95% CI = −0.34–0.66). Note that these analyses included data for subjects that dropped out before Appointment 4, because we deemed compliance to begin with splint delivery. Reasons for reduced compliance included forgetting to wear the splint, misplacing splint, and/or forgetting to take the splint on vacations (seven SOVA; seven MI). Issues with splint acceptance, for example, changes to the bite or splint being too tight, represented the remaining reasons (two SOVA; eight MI).

Figure 5 shows results for MGI (B) and PI (C), at baseline, Week 1 and Month 4. MI data are filled bars, SOVA data are open bars. No significant differences existed between subject groups for either index, (for MGI, F[1,53] = 1.213, *p* = .276, partial η^2^ = 0.022, 95% CI = 0–0.15; for PI, F[1,53] = 0.426, *p* = .517 partial η^2^ = 0.008, 95% CI undefined). There were significant differences across raters (for MGI, F[3,53] = 163.7, *p* < .001, partial η^2^ = 0.903, 95% CI = 0.84–0.93; for PI, F[3,53] = 2.943, *p* = .041, partial η^2^ = 0.143, 95% CI = 0–0.28), but there were no interactions between rater and subject group (for MGI, F[3,53] = 1.037, *p* = .384, partial η^2^ = 0.055, 95% CI = 0–0.16; for PI, F[3,53] = 0.904, *p* = .446, partial η^2^ = 0.048, 95% CI undefined).

Because all splints were maxillary, we hypothesized splints might impact maxillary periodontal health. Therefore, we evaluated within‐subject between‐arch effects. For MGI scores, there were no significant differences across the four areas, viz., BU, LU, BL, LL, (F[3,53] = 2.516, *p* = .060, partial η^2^ = 0.125, 95% CI = 0–0.26), nor did any of the four areas show significant between‐group differences (F[3,53] = 0.309, *p* = .819, partial η^2^ = 0.005, 95% CI undefined). There were no significant changes over the 4‐month time period in any of the four areas between groups (F[3,174] = 2.073, *p* = .106, partial η^2^ = 0.035, 95% CI = 0–0.09).

However, PI scores differed significantly within subjects across the four areas (F[3,174] = 17.15, *p* < .005, partial η^2^ = 0.228, 95% CI = 0.12–0.32). The PI was significantly lower for LU than for the other three areas (*p* < .05). Although the full model suggested significant differences between treatment groups by area (F[3,174] = 3.759, *p* = .016, partial η^2^ = 0.061, 95% CI = 0.003–0.13), pairwise comparisons did not reveal any significant differences (*p* > .098). No significant differences existed between groups by area through time (F[3,174] = 0.587, *p* = .589, partial η^2^ = 0.010, 95% CI undefined). Finally, no significant between‐group differences existed at baseline (Appointment 2) for measures of either MGI (U = 447, *p* = .799, d_Cohen_ = 0.067, 95% CI = −0.44–0.57) or PI (U = 433.5, *p* = .654, d_Cohen_ = 0.117, 95% CI = −0.39–0.62).

Figure 3b shows areas where splint wear was sampled; the means (1 *SD*) wear, in mm, on the splints over the 4‐month time period are shown in Figure 3c. There were no significant differences between groups (F[1,57] = 2.173, *p* = .146, partial η^2^ = 0.037, 95% CI = 0–0.17).

Finally, subjects were allowed to write open‐ended responses to the question, “What do you like best about your splint?” The main responses among MI splint subjects had to do with: (a) fit and comfort (N = 19/31), (b) protection of teeth from bruxism (N = 9/31), and (c) help with muscle relaxation (N = 3/31). The main responses among SOVA subjects had to do with: (a) fit and comfort (N = 20/30), (b) the splint's small size (N = 7/30), (c) protection of teeth from bruxism (N = 3/30), (d) help with muscle relaxation (N = 2/30), and (e) affordability (N = 1/30).

## 4 DISCUSSION

Ideally, participants randomly assigned to groups are well‐matched in RCT. We carefully matched groups based on gender and presence/absence of TMD signs/symptoms. Fortuitously, the groups were also closely matched across a number of other factors, including ethnicity, clinically‐observed tooth wear severity, self‐reported bruxing nights/week, and data from several surveys including the JFLS‐20, OBC, PSS, MOS, OHIP, TSK for TMD. There were also no significant between‐group differences for more detailed TMD findings as well (Table 3).

Perhaps the most significant finding was the inability of subjects to form the OTC splint according to instructions. Only 4/31 SOVA splints were clinically acceptable. All subjects were ultimately helped to fabricate clinically acceptable SOVA splints, but this would not occur routinely. It is highly likely that virtually any OTC appliances currently in use are being improperly fabricated. We strongly recommend that dental professionals play pro‐active, engaged roles with their patients who possess OTC appliances. It is noteworthy that, based on our findings, the SOVA splint is now available only through dentists and not available OTC.

This study was prompted by the fact that insurers often cover one splint per lifetime; however, rarely do splints last a lifetime. Because severe bruxers are more likely to require replacements, the unfortunate consequence is that patients who need the benefits the most stand to pay the most out‐of‐pocket. This suggests why OTC devices are becoming increasingly popular.

Clinical studies of obstructive sleep apnea (OSA) appliances emphasize the need to evaluate compliance and efficacy in terms of mean disease alleviation (Vanderveken et al., 2013) or effectiveness (Sutherland, Phillips, & Cistulli, 2015). One treatment may be more efficacious but have poorer compliance than another treatment.

But what defines efficacy in a SB oral appliance? The etiology and pathophysiology of SB are multifactorial and complex (Bader & Lavigne, 2000; Manfredini, Winocur, Guarda‐Nardini, Paesani, & Lobbezoo, 2013). Furthermore, oral appliances do not inhibit SB (Harada, Ichiki, Tsukiyama, & Koyano, 2006; Jagger, 2008). In this study, we operationally defined efficacy in terms of retention and stability, periodontal health, nocturnal EMG architecture, and structural integrity, that is, material wear. Periodontal problems were considered, due to the perforated design of the SOVA appliance.

The results found that efficacy was similar between the two devices in terms of stability, retention, periodontal health, and splint surface wear. On the other hand, there were significantly more Bh^−1^ and BE^−1^ in the SOVA group compared with the MI group, suggesting that the SOVA splint may exacerbate SB. Whether this is clinically significant remains unclear.

There are shortcomings with performing SB sleep studies with only EMG monitoring, cf. Castroflorio et al. (2015; Manfredini et al., 2014; Yachida et al., 2016). On the other hand, EMG monitoring of SB can be reliable (Castroflorio, Deregibus, Bargellini, Debernardi, & Manfredini, 2014; Gallo, Lavigne, Rompre, & Palla, 1997; Haketa et al., 2003; Minakuchi et al., 2012; Stuginski‐Barbosa, Porporatti, Costa, Svensson, & Conti, 2016). Given the precedent established in the literature, we would argue that the EMG results were worth reporting, but with emphasis on cautionary interpretation. Although we used audio as a means to improve our assessments, we found that, without video, sounds often remained ambiguous.

Of particular interest is the variable Bh^−1^, which was significantly different between groups, with mean (*SD*) of 18.8 (*SD* = 17.3) vs. 31.2 (*SD* = 4.24), for the MI versus SOVA group, respectively (Table 2). Doering et al. report 34.2 (*SD* = 10.6) Bh^−1^ in a SB population (Doering, Boeckmann, Hugger, & Young, 2008), which matches closely with our results. However, our results for Eh^−1^ (Table 2) was considerably higher than reported in either the Doering, et al. study (5.6 [*SD* = 1.3]) or Mayer et al., viz., 2–4 for mild to moderate SB and >4 for severe SB (Mayer, Heinzer, & Lavigne, 2016). This suggests that our Eh^−1^ values are over‐estimates as per (Manfredini et al., 2014). Whatever the case, our results suggest that something importantly different in SB architecture associated with SOVA versus MI splint wear may be occurring. This might be partly due to the softer material and larger occlusal contact areas provided by the SOVA versus MI splint. Further work needs to be done, ideally with PSG, to assess whether SOVA versus MI splints differentially impact SB architecture.

We developed a few novel methods to help evaluate splints, including motion analysis of stability, estimating bite forces during stability tests, and assessment of material wear. Motion analysis has an established record in the oral motor literature (Gerstner, Lafia, & Lin, 2005; Gerstner, Marchi, & Haerian, 1999; Gerstner & Parekh, 1997; Tanaka, Yamada, Maeda, & Ikebe, 2016; Wilson, Luck, Woods, Foegeding, & Morgenstern, 2016), and so we are reasonably confident that our stability results are objective and accurate. Similarly, previous bite force estimates using EMG data have used variables similar to ours (Gonzalez et al., 2011; Van Eijden, Brugman, Weijs, & Oosting, 1990). Thus, the stability results are probably reasonable.

A digital method of evaluating splint wear was developed by Korioth et al. (1998) similar to ours. The wear seen in the previous study was also low. It is possible that the 4‐month time period does not provide sufficient time to identify wear, let alone estimate wear rates. Longer‐term studies will likely be more revealing.

We measured compliance through self‐report and found no significant between‐group differences. Evidence suggests that self‐report is a reasonable compliance measure, even when compared to embedded micro‐sensor methods (Vanderveken et al., 2013). Based on patient satisfaction, there did not seem to be any differences between groups, and reasons for not wearing splints were also similar between groups. Thus, the similar scores for both satisfaction and compliance suggest no significant differences in splint preference.

Several study limitations existed. Firstly, the study was not double‐blinded. This would have been difficult to do, given the distinct fabrication methods and appearances of each splint (Figure 2). Obviously splint fabrication assessment could not be blinded either. However, single blinding was done for virtually all other study aspects, for example, stability, retention, periodontal assessment, EMG analysis, compliance tabulation, and statistical analyses. Also, where multiple investigators were involved, we allotted equal numbers of subjects from each group to each investigator, thereby reducing the impact of investigator biases on results.

Another study limitation was our use of subjects with “probable” as opposed to “definite” diagnoses of SB (Lobbezoo et al., 2013). Given that dentists do not routinely obtain sleep studies on patients with SB, dentists usually treat patients with “probable” SB diagnoses anyway, and in this respect our subjects probably represent the “typical” population treated by dentists for SB. We recognize that this limitation may be a reason for the lack of observed splint surface wear. On the other hand, results of the nocturnal EMG study suggest active SB habits in our subjects.

Other study limitations include the small sample sizes and the abbreviated time period over which the study was performed. Many of the effect sizes demonstrated fairly large confidence intervals, which is likely due to the small study size. Future, larger projects may consider cross‐over designs, inclusion of PSG, longer‐term splint wear, and use of compliance sensors, among other things.

In conclusion, because OTC splints were difficult to construct, we highly recommend that OTC splints be monitored by dentists. We recognize the need for inexpensive alternatives. Ideally, a large RCT would demonstrate definitely whether an OTC splint would be a legitimate solution. This would potentially provide an important measure of external validity. It is unlikely that such a large RCT will occur in the near future. Hence, a practical approach would be for dentists to be vigilant with OTC splint fabrication and use, since this appears to be a practical alternative for at least the near future.

## CONFLICT OF INTEREST

The authors have no conflicts to report.

## Supporting information


**Appendix S1.** Supporting Information.
